# Conformational preferences of α-fluoroketones may influence their reactivity

**DOI:** 10.3762/bjoc.13.284

**Published:** 2017-12-29

**Authors:** Graham Pattison

**Affiliations:** 1 Department of Chemistry, University of Warwick, Gibbet Hill Road, Coventry CV4 7AL, UK

**Keywords:** α-halogenated ketones, conformational analysis, reactivity, stereoelectronic effects

## Abstract

Fluorine has been shown in many cases to impart specific and predictable effects on molecular conformation. Here it is shown that these conformational effects may have an influence on reactivity through studying the relative reactivity of various α-halogenated ketones towards borohydride reduction. These results demonstrate that the α-fluoro ketones are in fact a little less reactive than the corresponding α-chloro and α-bromo derivatives. It is suggested, supported by computation, that this effect is due to reactive conformations in which the C–X bond is orthogonal to the carbonyl group for good orbital overlap being disfavoured in the case of fluoro ketones.

## Introduction

α-Halogenated ketones are widely used electrophiles in organic synthesis, being highly reactive in both nucleophilic addition to the carbonyl group and in S_N_2 nucleophilic displacements [1]. Our research group has recently been exploring the synthesis and reactivity of α-fluorinated ketones [2–4] and here their reactivity relative to other halogenated ketones is compared.

It is well established that the halogen leaving groups in these substrates are highly activated by orbital overlap with the adjacent carbonyl group, making α-halogenated ketones one of the most reactive classes of electrophiles available to synthetic chemists for S_N_2 substitution [5]. The orbital overlap in α-halogenated ketones also provides activation to the carbonyl group, making it more reactive towards nucleophilic addition than non-halogenated carbonyl compounds [6]. However, relatively little work has been performed previously to quantify the effects that different α-halogen atoms have on carbonyl reactivity.

This paper aims to examine some of the effects that α-halogenation can impart on carbonyl reactivity with a particular emphasis on the effects of α-fluorination. As the most electronegative element, fluorine is often involved in introducing unusual properties to organic molecules, whether by its strong inductive effect, interactions of its tightly-held lone pairs or through the strong dipole moment it can induce in molecules [7].

To begin to quantify the effects of α-halogenation on carbonyl reactivity we wished to measure the relative reactivity of various α-halogenated ketones towards nucleophilic addition. As these are highly reactive systems obtaining rate profiles can be difficult due to the short time-scales for measurements, so instead relative reactivity was measured through a series of competition experiments. A competition experiment between two substrates stopped at low conversion (<20%) provides a good approximation for the relative initial rates of reaction of the two substrates through measurement of the relative amounts of the two products formed.

These competition reactions should proceed cleanly, with minimal byproduct formation, and in the case of examining the reactivity of the carbonyl group of α-halogenated ketones, should show very high regioselectivity for nucleophilic addition to the carbonyl group rather than nucleophilic displacement of the halogen atom. Another important consideration in this scenario is that the nucleophilic addition to the carbonyl group should not be reversible. The choice of nucleophile for study should take all of these important considerations into account. The nucleophilic addition of sodium borohydride to various α-halogenated ketones was therefore chosen for examination as borohydride addition is irreversible and shows a very high preference for direct addition to the carbonyl group.

## Results and Discussion

**Scheme 1:**
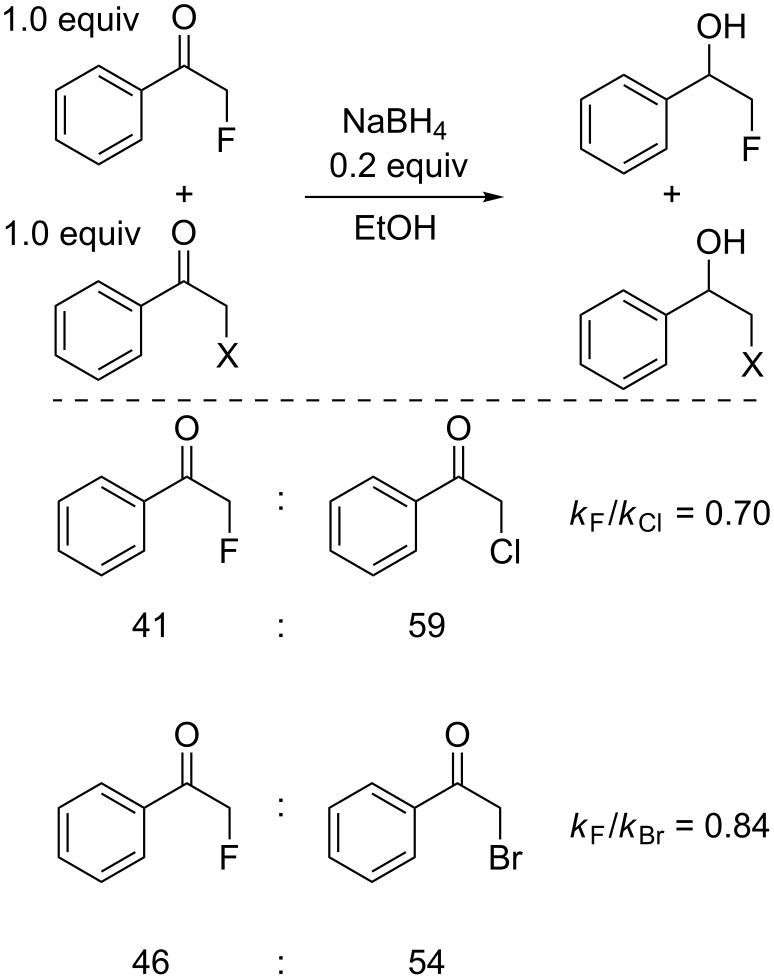
Relative reactivity of α-fluoroacetophenone to α-chloroacetophenone and α-bromoacetophenone.

The initial focus of this work was on comparing the reactivity of various α-monohalogenated ketones to examine the effects of different halogen atoms on the reactivity. The reactivity of α-fluoroacetophenone was compared to α-chloro- and α-bromoacetophenone in sodium borohydride reductions, using 0.2 equiv of NaBH_4_ to 1.0 equiv of α-fluoroacetophenone and 1.0 equiv of the second α-haloacetophenone to ensure the reaction stopped at low conversion. The relative ratios of reduced products were then compared using ^1^H NMR spectroscopy (Scheme 1). All results are the average of at least two repetitions, with the NMR integrals, set to the fluorinated peak equal to 1.00, consistent to at least ±0.1.

**Scheme 2:**
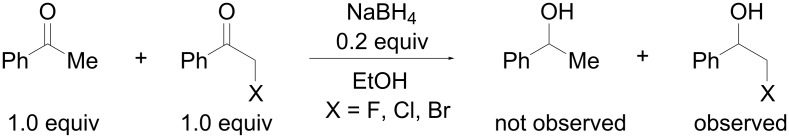
Competitive reduction of haloacetophenones and acetophenone.

Interestingly, both experiments showed α-fluoroacetophenone to be less reactive than both α-chloroacetophenone and α-bromoacetophenone, with a slightly larger difference in reactivity for α-chloroacetophenone. The reactivity of α-iodoacetophenone was not examined as it proved to be unstable under the reaction conditions. This higher reactivity of the non-fluorinated ketones was not the expected outcome through simple arguments of electronegativity differences. Comparison of the reactivity of each α-haloacetophenone to non-halogenated acetophenone showed the halogenated derivatives to be significantly more reactive (no reduction of acetophenone could be observed) (Scheme 2).

**Figure 1:**
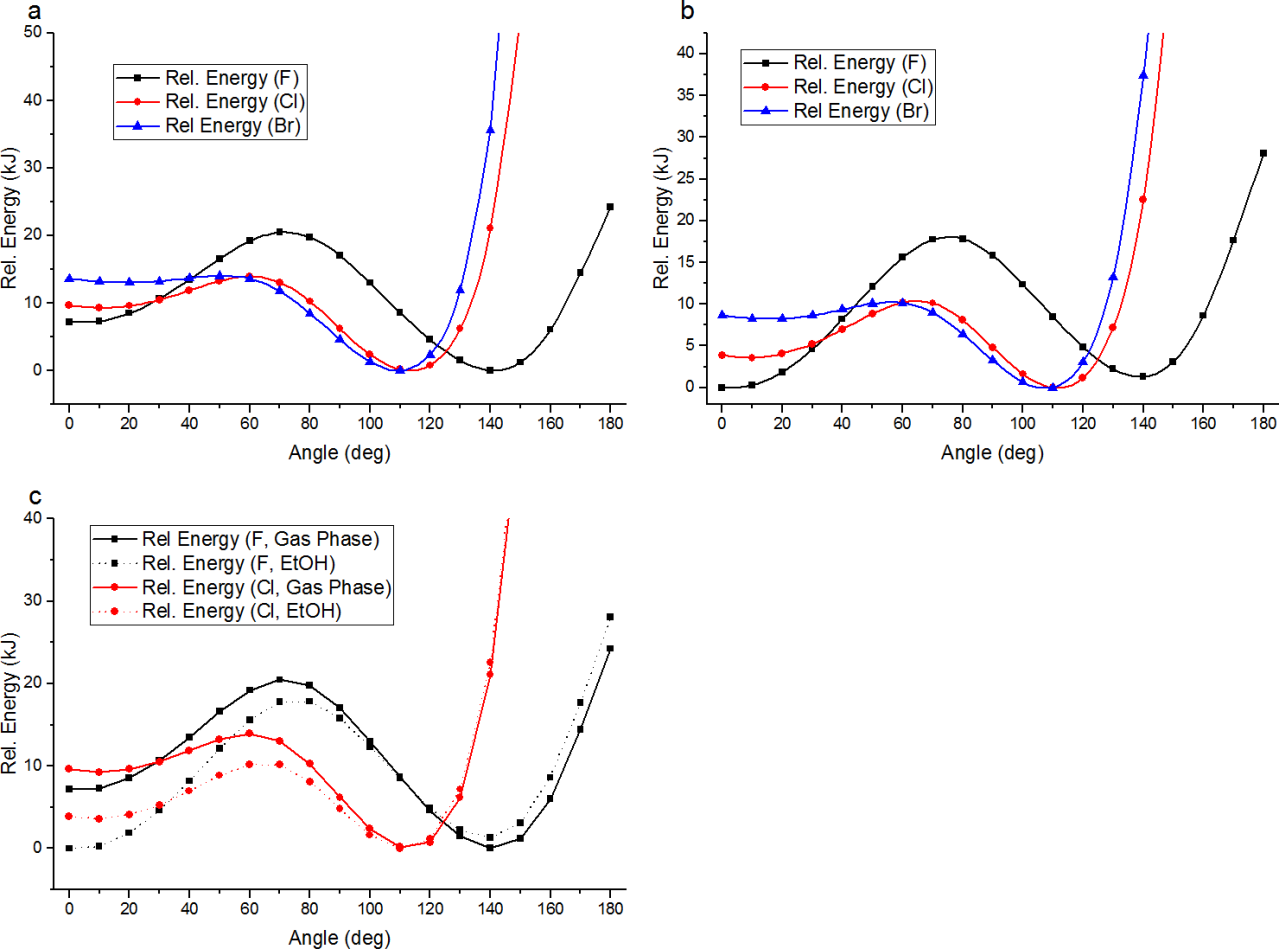
Conformational energy profiles of halogenated acetophenones (a) in gas phase; (b) in EtOH; (c) overlay of gas phase and EtOH for F and Cl.

Potential reasons behind the lower than expected reactivity of α-fluoroacetophenone were then considered. It is known that fluorine can dramatically influence the conformational preferences of molecules [8–9], so began by simulating the conformational energy profile of each α-haloacetophenone, calculating the energy of each compound as the carbon–halogen bond is rotated through 10° increments in both the gas phase and in ethanol as reaction solvent (Figure 1) [10].

**Figure 2:**
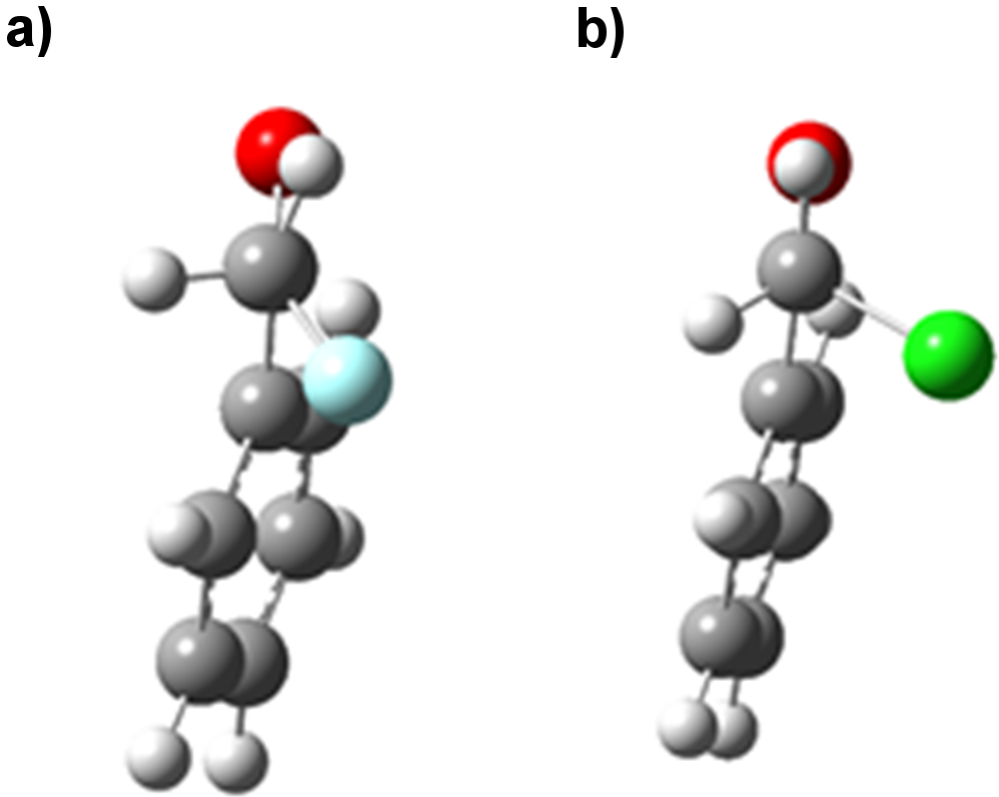
Optimised gas phase geometries of (a) α-fluoroacetophenone and (b) α-chloroacetophenone emphasising the smaller dihedral angle preferred by the chlorinated derivative.

**Figure 3:**
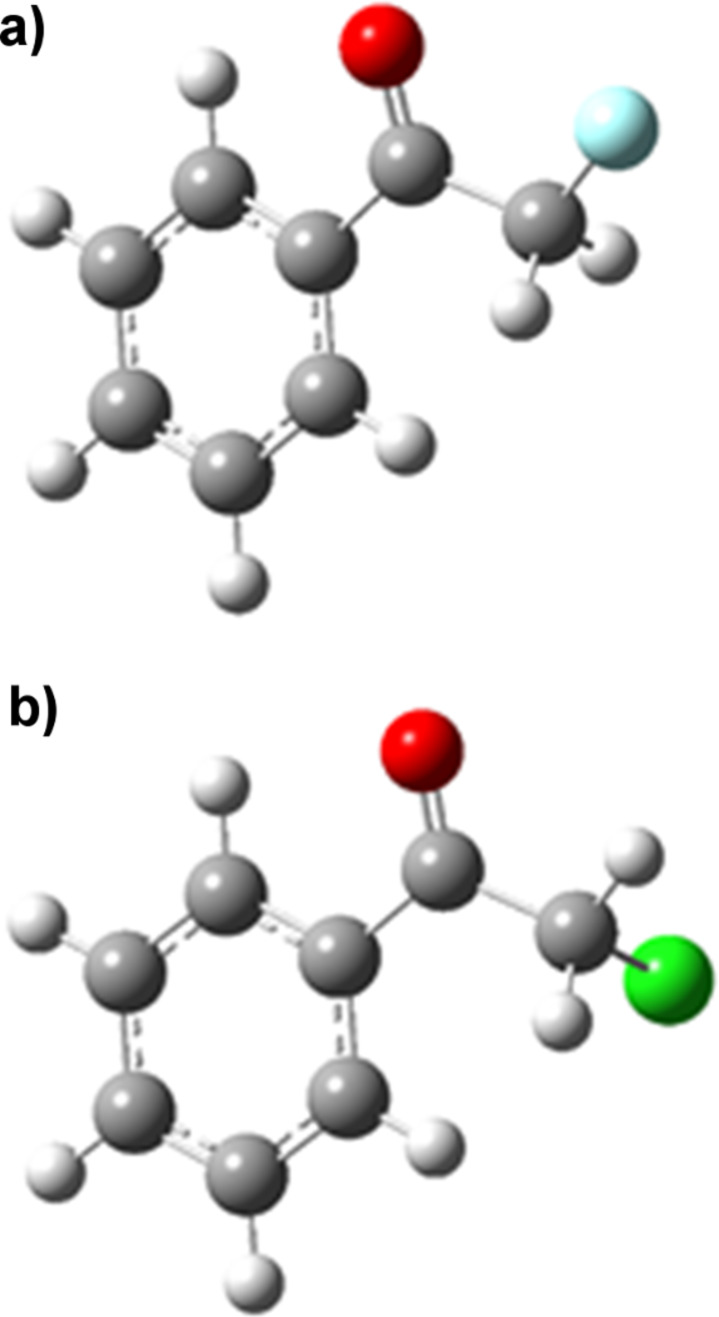
Most stable conformations of (a) α-fluoroacetophenone and (b) α-chloroacetophenone in ethanol.

The fluorinated acetophenone showed significant differences in conformational energy to the chlorinated and brominated variants. The energy minimum for α-fluoroacetophenone was displayed at an O=C–C–X dihedral angle of around 140° in the gas phase, whilst the chloro- and bromoacetophenones both showed minima around 110°. Highly polar conformations which place the C–X bond in the same plane as the carbonyl group were favoured in the polar solvent ethanol; indeed in ethanol the lowest energy conformation of α-fluoroacetophenone is calculated be a *cis*-conformation with a O=C–C–X dihedral angle of 0°. Figure 2 shows equivalent 3-dimensional views along the C–C bond between the carbonyl group and C–X bond emphasising the smaller dihedral angle preferred by the chlorinated derivative in the gas phase. Figure 3 compares the lowest energy conformations of α-fluoroacetophenone and α-chloroacetophenone in the polar solvent ethanol. Experimental work by Olivato amongst others supports these conformational preferences [11–15]. IR spectroscopy was used to show an increased preference for a *cis* (0° dihedral angle) compared to a *gauche* (150°) conformation in α-fluoroacetophenone compared to α-chloroacetophenone [16].

**Figure 4:**
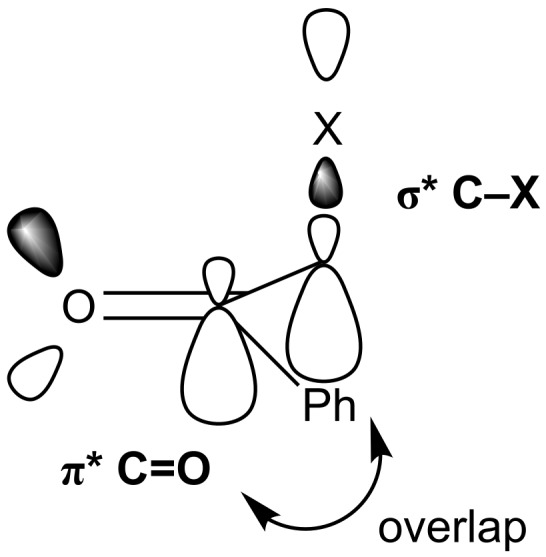
Expected reactive conformation of halo-acetophenones.

This has significant implications for the orbital overlap in these systems as it would be expected that the best orbital overlap between the C=O π* orbital and C–X σ* orbital which is necessary for high reactivity would be achieved when the O=C–C–X dihedral angle is 90° (Figure 4). Previous calculations by Paddon-Row on nucleophilic additions to fluoroethanal and 2-fluoropropanal have suggested that additions to this conformation lead to the most stabilized transition state [17], whilst experimentally, nucleophilic addition of NaBH_4_ to 2-fluoropropiophenone leads to the *anti*-diastereoisomer that would be expected by polar Felkin–Anh addition to this conformation [18]. However, around a 90° dihedral angle in the conformational energy profiles, the fluorinated derivative is around 10 kJ·mol^−1^ higher in relative energy than the brominated and chlorinated analogues, suggesting that it will be energetically unfavourable for α-fluoroacetophenone to access these particularly reactive conformations.

**Figure 5:**
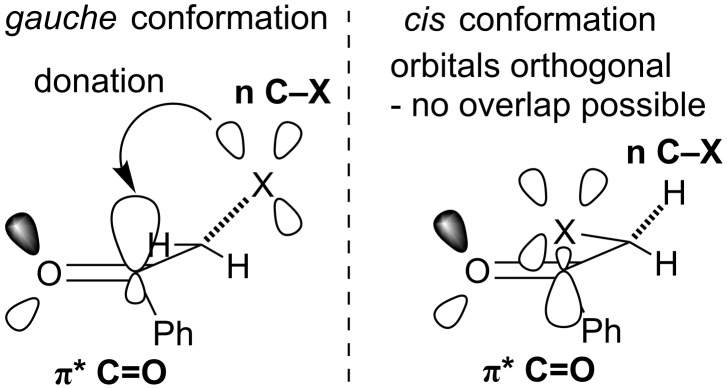
Orbital interactions in *gauche*- and *cis*-conformations of haloacetophenones.

Orbital interactions with the C=O π* orbital are possible at dihedral angles other than 90°. For example in a *gauche* conformation (120–150° O=C–C–X dihedral angle), overlap between the halogen atom lone pairs and the C=O π* orbital is possible, weakening the π-bond and increasing the reactivity towards nucleophilic attack (Figure 5). It would be expected that of all the halogens, fluorine’s lone pairs would overlap most strongly with the carbonyl π*-orbital and decrease its bond order. However, particularly in polar solvents like ethanol, it is the *cis* conformation (0° O=C–C–X dihedral angle) which is preferred for α-fluoroacetophenone, which places the C–F bond in the same plane as the C=O bond, making orbital interactions impossible. Although orbital interactions between chlorine’s lone pairs and the C=O π* orbital are expected to be weak, at least α-chloroacetophenone has a lowest energy *gauche* conformation where these orbital interactions are possible, which may provide some degree of electronic activation.

The variability of the relative reactivity of α-fluoroacetophenone and α-chloroacetophenone with temperature were then investigated (Table 1). The same methodology using competition experiments stopped at low conversion was used at 20 °C temperature increments from 0 to 60 °C. This showed an increase in the relative reactivity of the fluorinated derivative as the temperature was increased. One potential reason for this is that increased conformational freedom at higher temperatures makes more reactive conformations more accessible to the fluorinated acetophenone.

**Table 1:** Relative reactivity of α-fluoroacetophenone and α-chloroacetophenone at different temperatures.

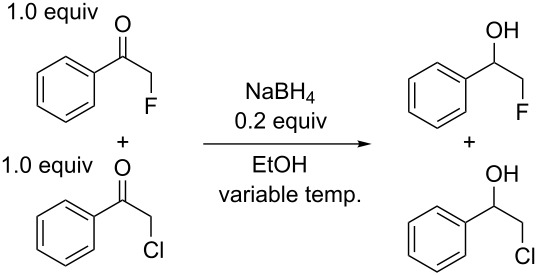	

Temperature [°C]	*k*_F_/*k*_Cl_

0	0.58
20	0.70
40	0.77
60	0.86

**Figure 6:**
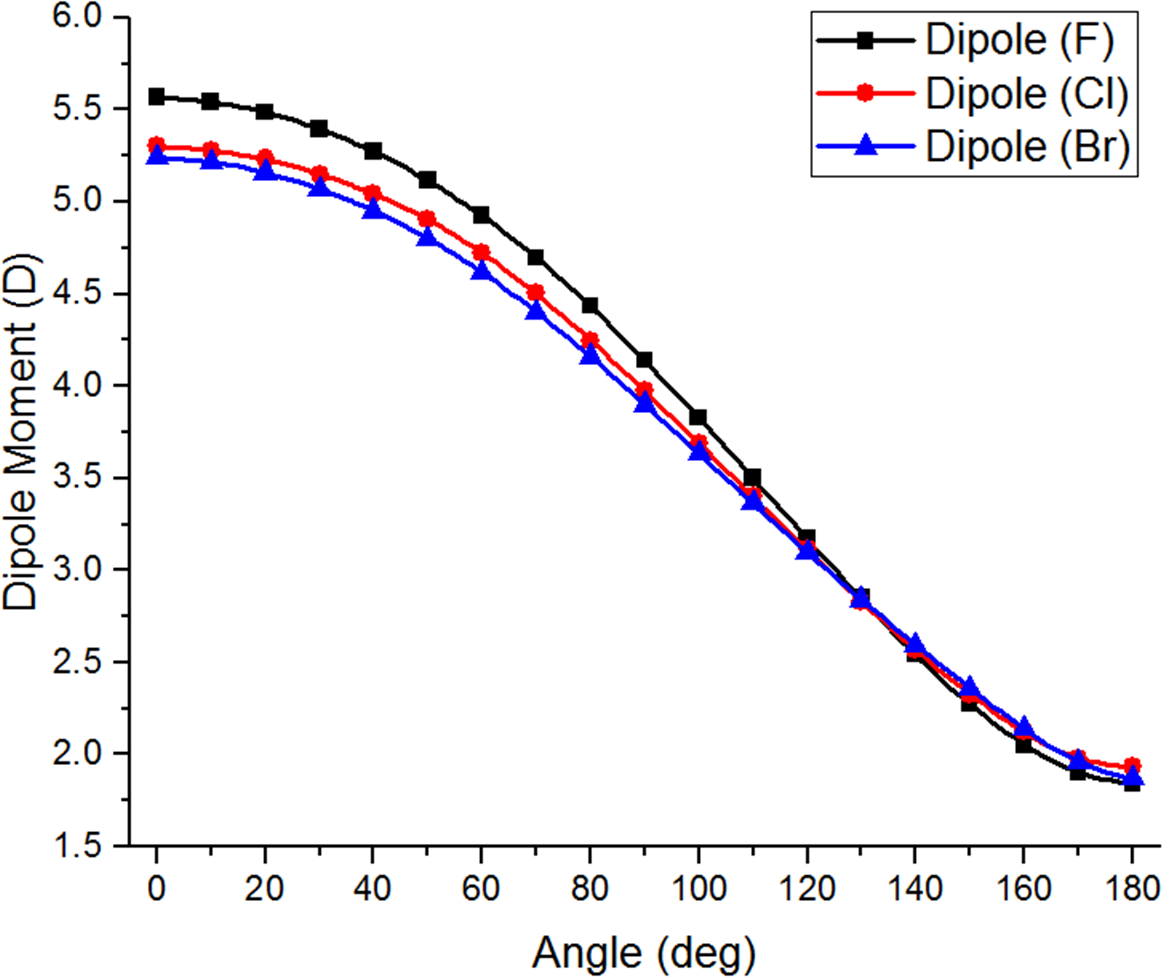
Variation of dipole moment with angle for haloacetophenones.

Potential reasons for the different conformational preferences of the α-halogenated acetophenones were then examined. One possibility is that the increased electronegativity of fluorine induces a high dipole moment at small O=C–C–X dihedral angles and that therefore larger dihedral angles are favoured as this minimizes the molecule’s overall dipole moment. However, computational analysis of the angular variation of the dipole moment of each α-haloacetophenone did not show a significant variation between the different halogens (Figure 6).

**Figure 7:**
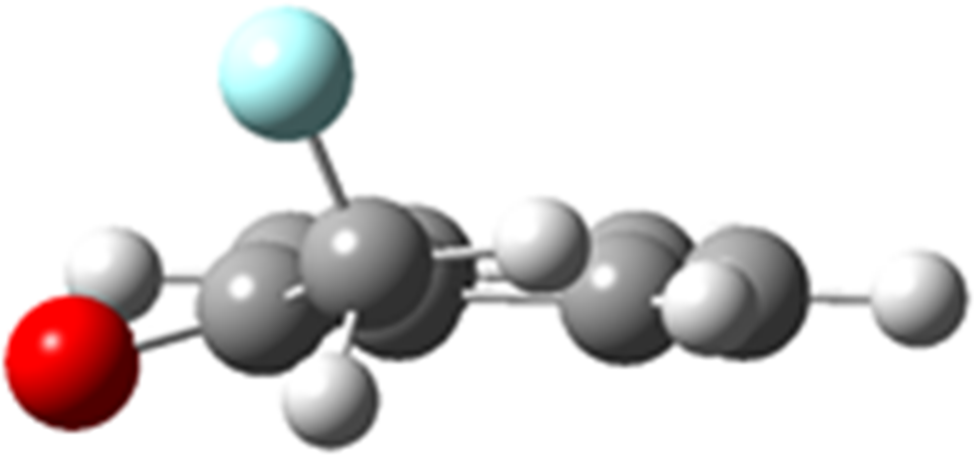
Highest energy conformation of fluoroacetophenone, emphasizing the closeness of approach of fluorine atom to carbonyl π-orbital.

The highest energy conformations of α-haloacetophenones have a O=C–C–X dihedral angle of 60–70° and place the C–X bond roughly aligned with the π-system of the carbonyl group and aromatic ring (Figure 7). It may well be that in this conformation there is significant repulsion between the halogen lone pairs and the filled C=O π-orbital. The higher polarizability of higher halogens such as chlorine and bromine may be able to reduce this repulsion, however, the tightly held, non-polarizable lone pairs of fluorine are likely to experience this repulsive effect most strongly. The shorter C–F bond length may also play a role in this interaction, placing the fluorine atom closer to the carbonyl group. This will disfavour these conformations in the fluorinated derivatives, which also happen to be the most reactive conformations.

**Scheme 3:**
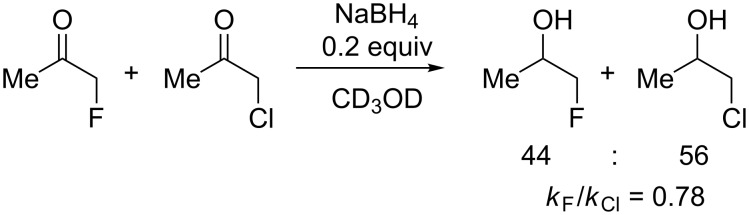
Competitive reduction of fluoroacetone and chloroacetone.

We then wanted to establish whether this lower reactivity of α-fluoro ketones compared to α-chloro ketones was transferable to other systems than acetophenones, and chose to compare the reactivity of fluoroacetone and chloroacetone (Scheme 3). The higher volatility of the reduced products in this case meant the reactions were performed directly in deuterated methanol before taking NMR of the reaction mixture without isolation.

**Figure 8:**
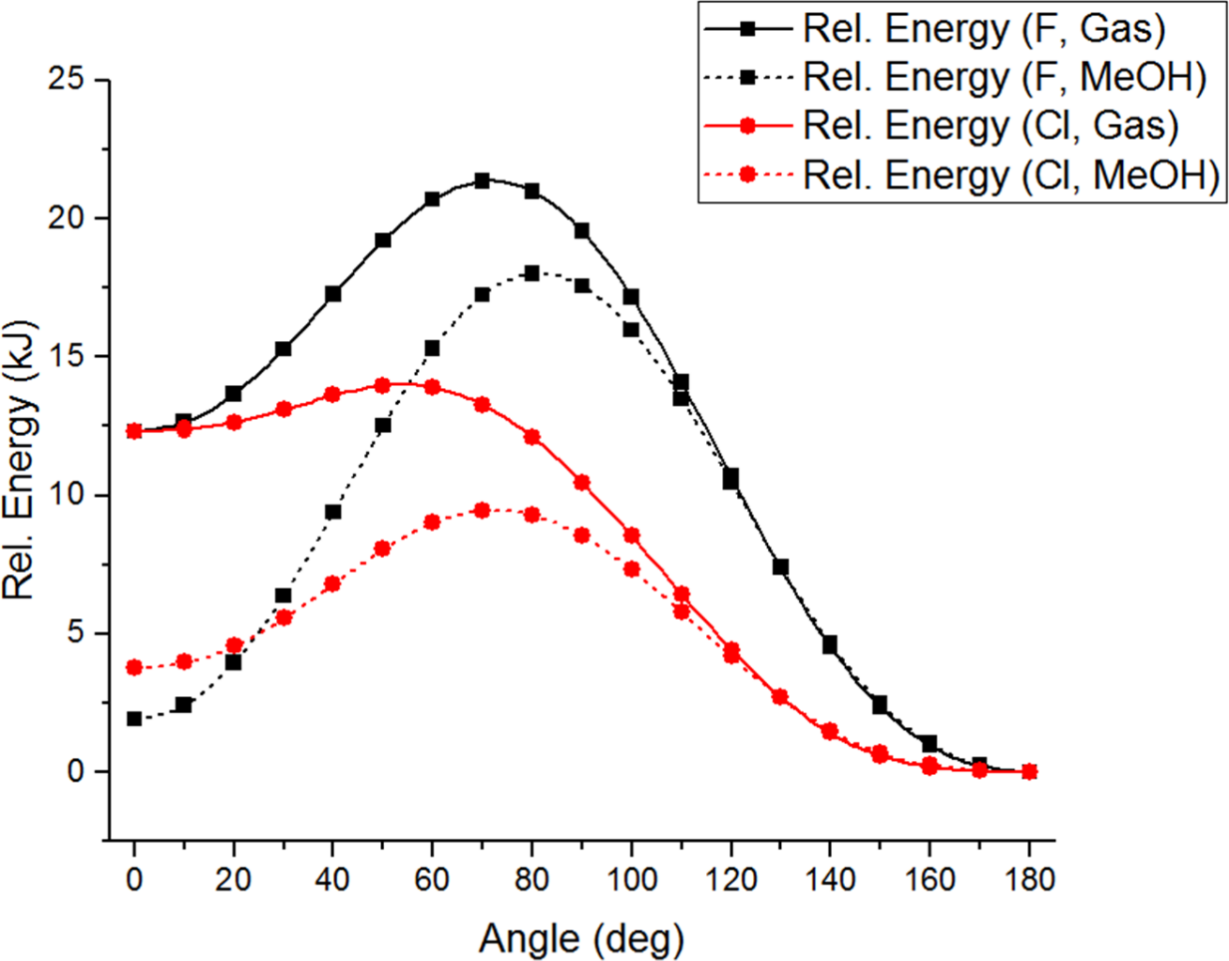
Conformational energy profiles of halogenated acetones in gas phase and in MeOH.

This again showed the α-fluoroacetone to be slightly less reactive than the α-chloroacetone. A similar conformational analysis of the bond rotation of the O=C–C–X dihedral angle for the chloro and fluoro derivatives was performed (Figure 8). This showed that, whist both molecules were most stable in an *anti*-conformation [19–21], the barrier to rotation of fluoroacetone was significantly higher than of chloroacetone, and the reactive conformations in which the halogen was orthogonal to the carbonyl group for C–X σ*/C=O π* overlap were significantly higher in energy for the fluorinated derivative. This offers further support to the theory that this may be a significant factor in slightly reducing reactivity of the fluorinated system relative to the chlorinated.

Again, as for the haloacetophenones, the *cis*-arrangement was significantly stabilized in methanol, particularly for fluoroacetone, although in this case a *trans*-arrangement was still more stable. Neither *cis*- nor *trans*-arrangements of the C=O and C–X bonds can offer any stabilization by donation of halogen lone pairs into the C=O π* orbital, so this orbital interaction is not relevant in the case of haloacetones. This conformational analysis is supported by previous work by Abraham and Rittner who used NMR coupling constants and theory to demonstrate that a *trans*-conformation of fluoroacetone is always most favourable, but that the energy difference to the *cis*-conformation decreases on solvation [22]. Work on related halo-acetaldehyde systems suggested that steric repulsions were the key contributing factor in determining these preferred conformations [23].

**Figure 9:**
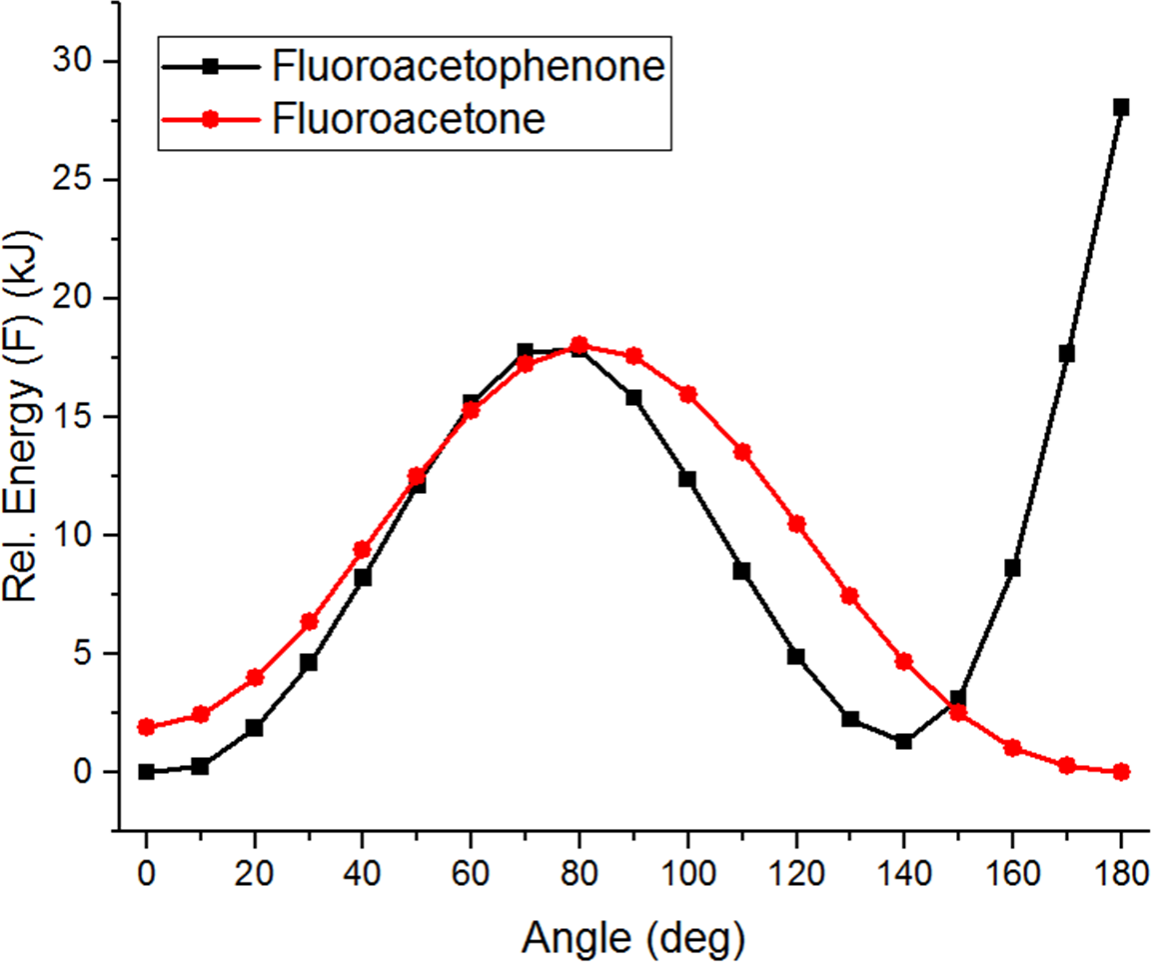
Overlay of conformational energy profiles of fluoroacetone and fluoroacetophenone.

Finally, the conformational profiles of fluoroacetone and fluoroacetophenone were compared by overlaying on the same graph (Figure 9). This showed a similar maximum energy for both, around the same angle, supporting the hypothesis that this is due to repulsion of fluorine lone pairs with the carbonyl π-system. Between 80° and 140° dihedral angles fluoroacetophenone is stabilized relative to fluoroacetone, likely due to overlap between the carbonyl C=O π-orbital and the aromatic ring π-system beginning to develop. However, at high dihedral angles (150–180°) fluoroacetophenone is significantly destabilized, likely due to steric interactions between the fluorine atom and *ortho*-hydrogens of the aromatic ring.

## Conclusion

The relative reactivity of various halogenated ketones in borohydride reduction have been studied, which established that the fluorinated derivatives display slightly lower reactivity than the chlorinated and brominated derivatives. This is the opposite that would be expected from simple electronegativity arguments and can be potentially explained by the higher energy barrier in the fluoro ketones to access reactive conformations which place C–X and C=O bonds at 90° to each other for optimal orbital overlap. The reason for this higher energy barrier in the fluorinated derivatives compared to other halogenated ketones is not fully understood, although could be related to repulsion between fluorine’s lone pairs and the carbonyl π-system, which will be reduced for other halogens due to their higher polarizability. A final factor which may explain the unexpectedly lower reactivity of fluorinated ketones is that they show a high preference in polar solvents to attain a *cis*-conformation, which place C=O and C–F bonds in the same plane and unable to undergo favourable orbital interactions.

## Experimental

NMR analysis was performed on a Bruker Avance III HD-400 system. Computational calculations were performed using the Gaussian-03 package using a MP2/6-311G++(d,p) basis set.

### Procedure for competition experiments

**Acetophenones.** A mixture of 2-fluoroacetophenone (69.1 mg, 0.5 mmol) and either 2-chloroacetophenone (77.3 mg, 0.5 mmol) or 2-bromoacetophenone (99.5 mg, 0.5 mmol) was dissolved in ethanol (1 mL) and heated/cooled to the appropriate temperature. Sodium borohydride (3.8 mg, 0.1 mmol) was added and the mixture stirred for 15 minutes. After this period HCl (1 M, 1 mL) was added, followed by diethyl ether (2 mL). The organic layer was separated, dried over MgSO_4_ and evaporated. ^1^H NMR in CDCl_3_ was measured of this crude mixture.

**Acetones.** A mixture of chloroacetone (46.3 mg, 0.5 mmol) and fluoroacetone (38.0 mg, 0.5 mmol) was dissolved in CD_3_OD at room temperature. Sodium borohydride (3.8 mg, 0.1 mmol) was added and the mixture stirred for 15 minutes. ^1^H NMR was measured of this crude mixture.

## Supporting Information

File 1 Copies of NMR spectra showing ratios of fluorinated and halogenated products.

File 2 Details of computational conformational analysis.
