# Supplementary material for: Conformational preferences of α-fluoroketones may influence their reactivity
Source: Beilstein J Org Chem. 2017 Dec 29;13:2915–21. doi: 10.3762/bjoc.13.284 (PMC5753061; doi:10.3762/bjoc.13.284)

Supporting Information

for

Conformational preferences of α-fluoroketones may influence their reactivity

Graham Pattison*

Address: Department of Chemistry, University of Warwick, Gibbet Hill Road, Coventry CV4 7AL, UK

Email: Graham Pattison - graham.pattison@warwick.ac.uk

* Corresponding author

Copies of NMR spectra showing ratios of fluorinated and halogenated products

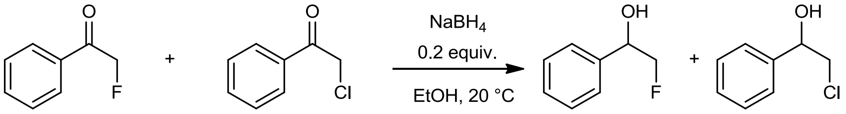

1.0 equiv. 1.0 equiv. 41 : 59

$k_F / k_{Cl} = 0.70$

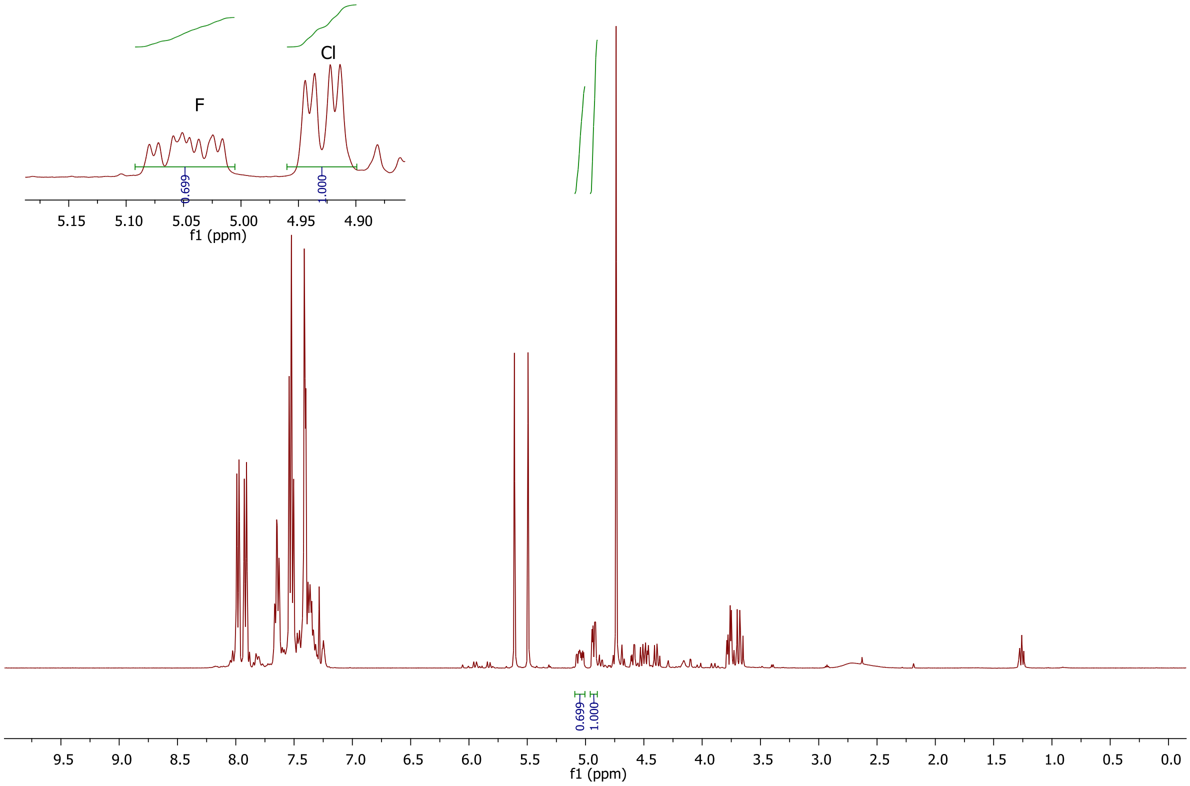

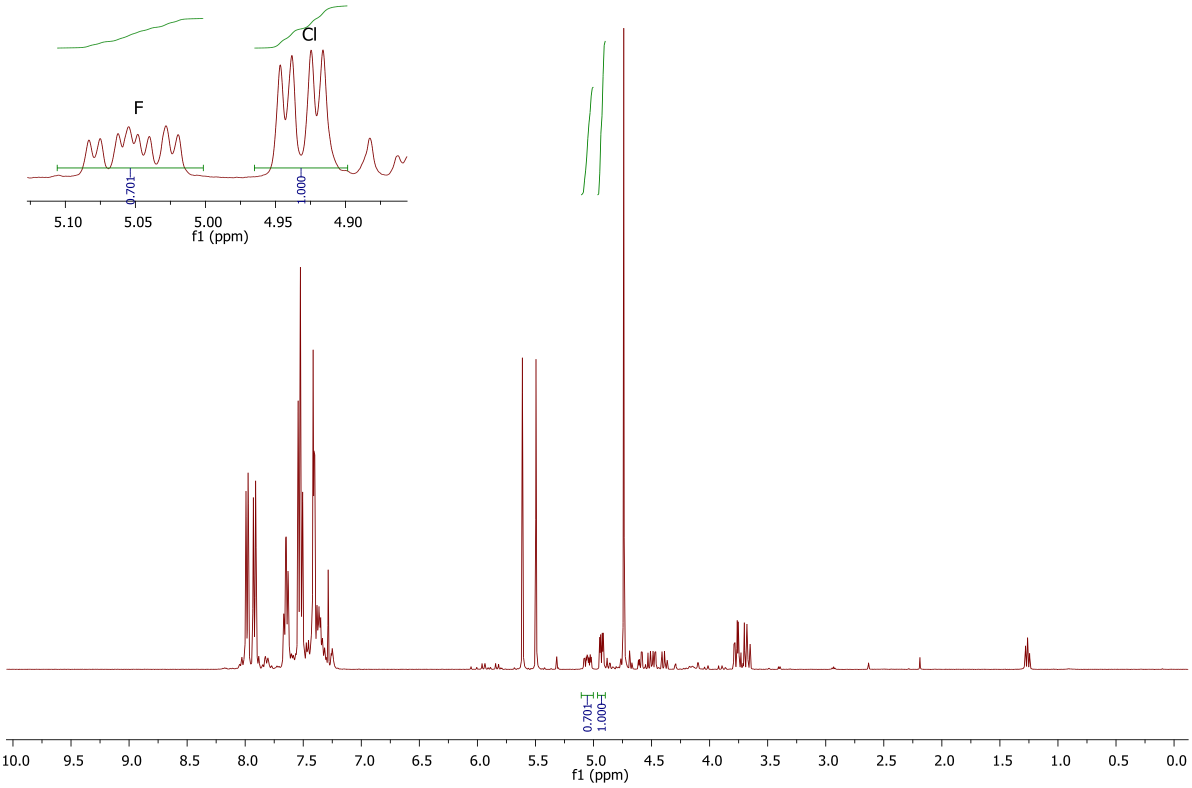

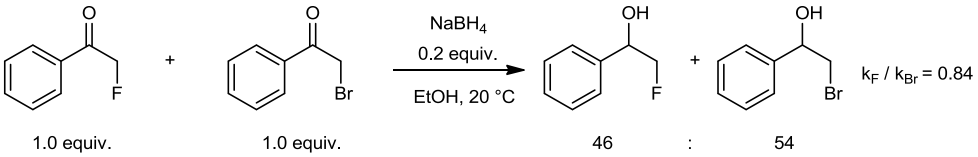

NaBH4
0.2 equiv.
EtOH, 20 °C
1.0 equiv.
1.0 equiv.
46 : 54
kF / kBr = 0.84

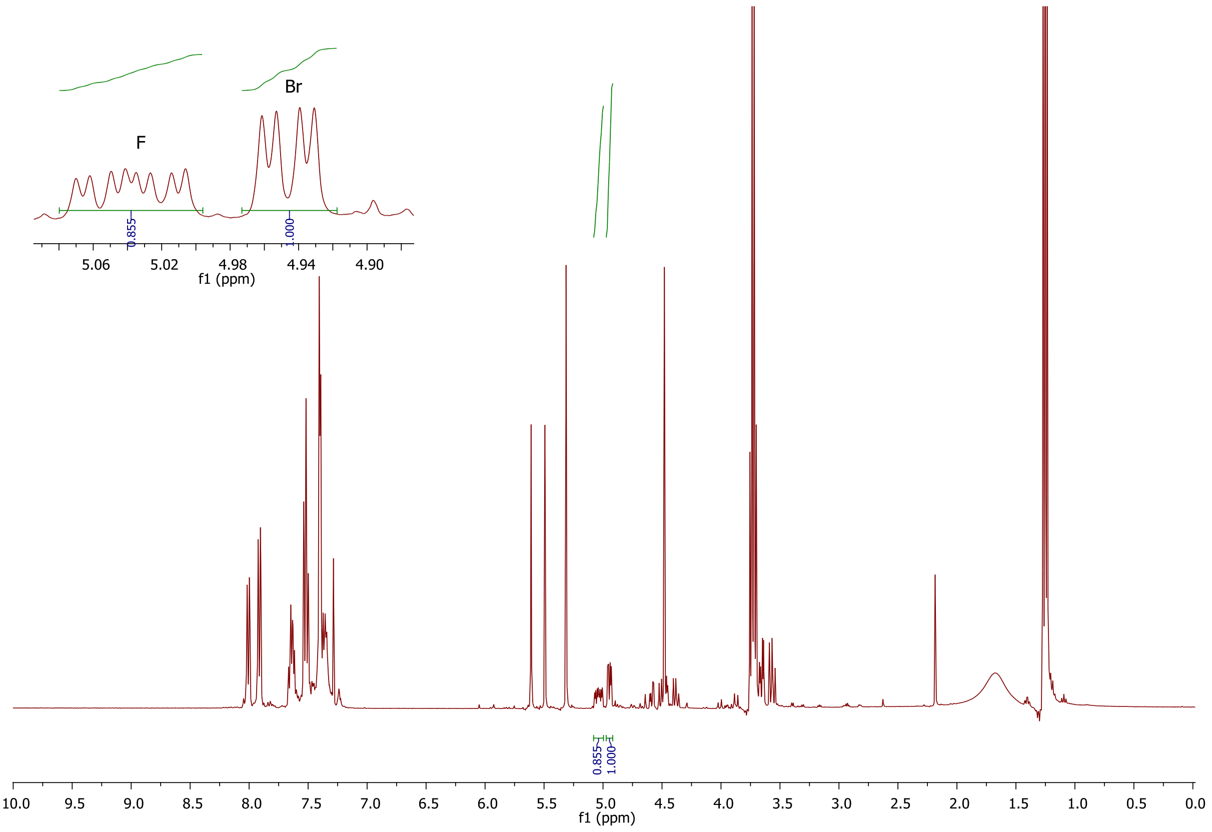

F
Br
0.855
1.000
f1 (ppm)

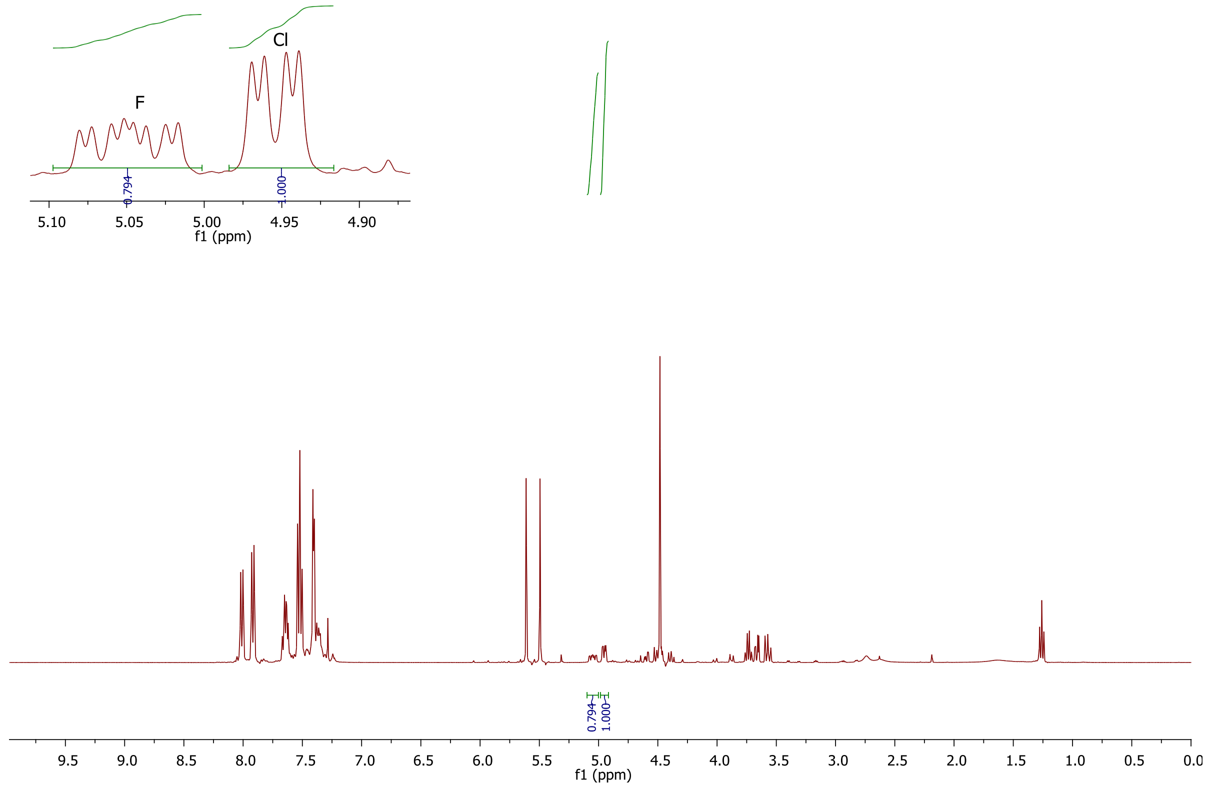

F
Cl
0.794
1.000
f1 (ppm)

0 °C

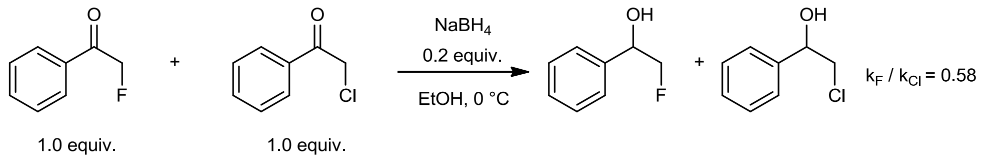
NaBH$_4$
0.2 equiv.
EtOH, 0 °C
1.0 equiv.
1.0 equiv.
k_F / k_{Cl} = 0.58

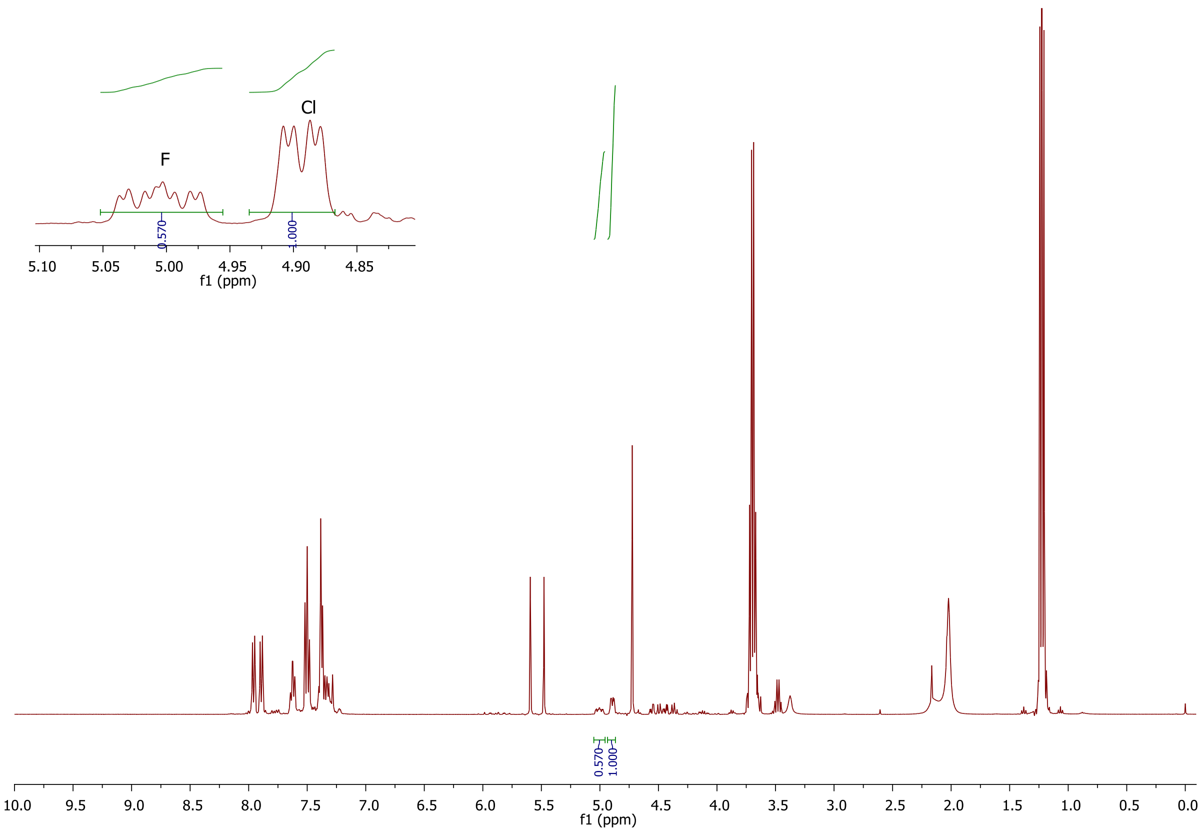
F
Cl
0.570
1.000
f1 (ppm)

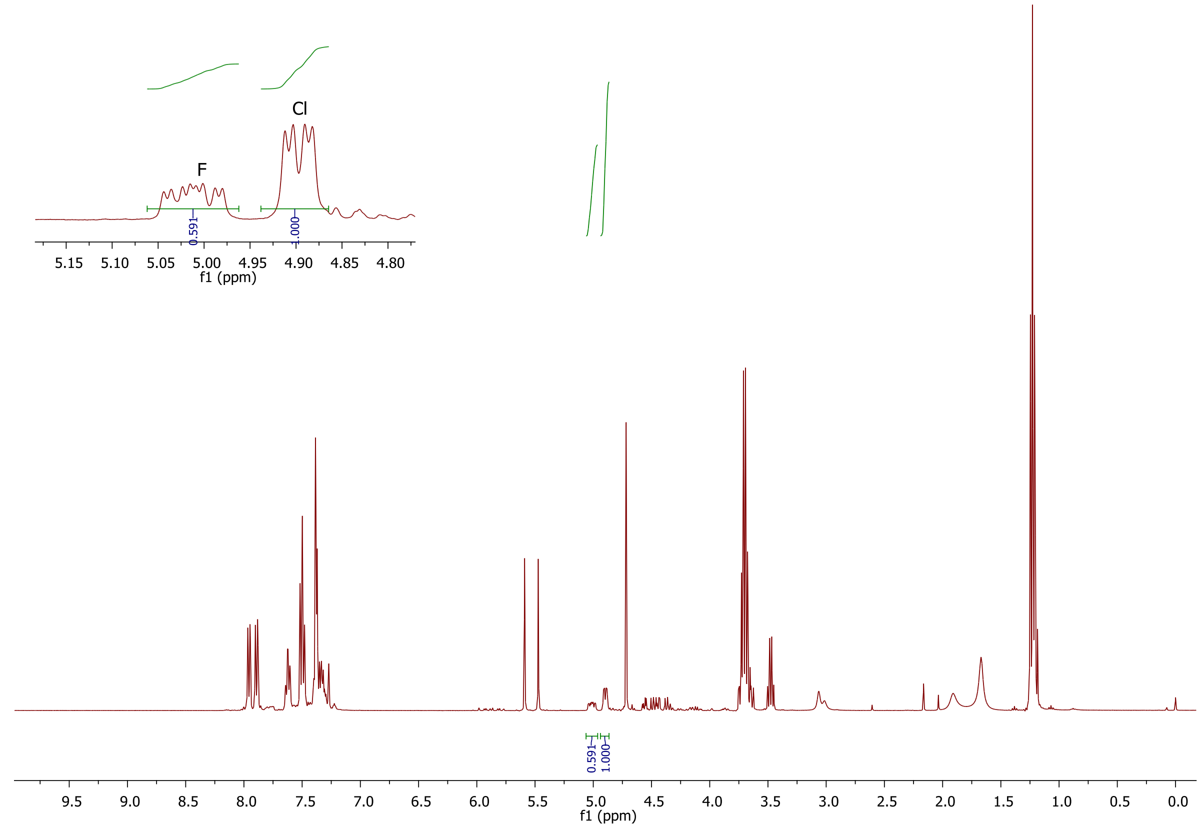
F
Cl
0.591
1.000
f1 (ppm)

40 °C

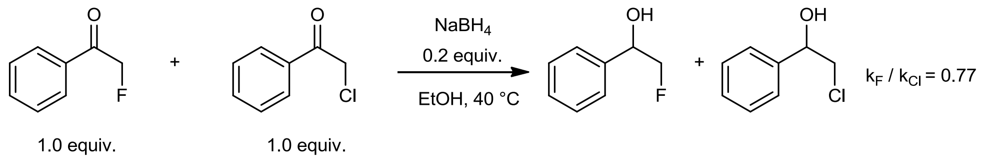
NaBH4
0.2 equiv.
EtOH, 40 °C
1.0 equiv.
1.0 equiv.
kF / kCl = 0.77

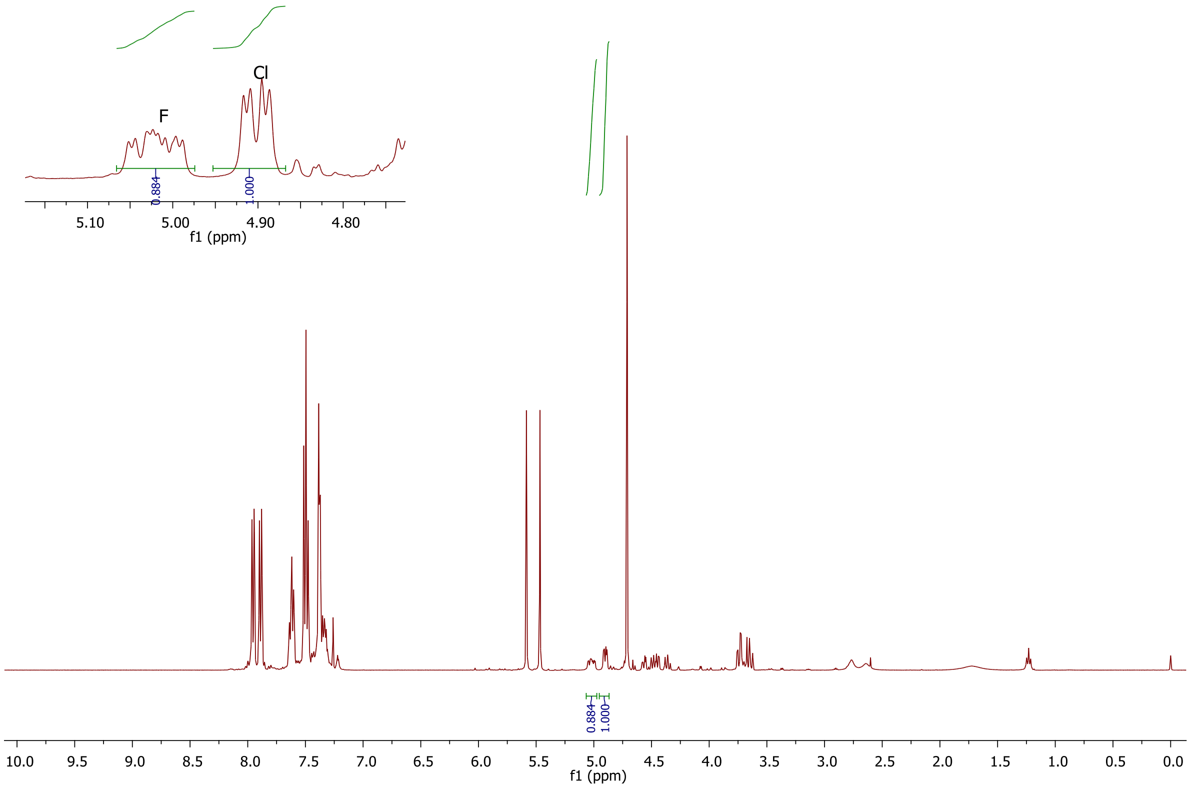
F
Cl
0.884
1.000
f1 (ppm)

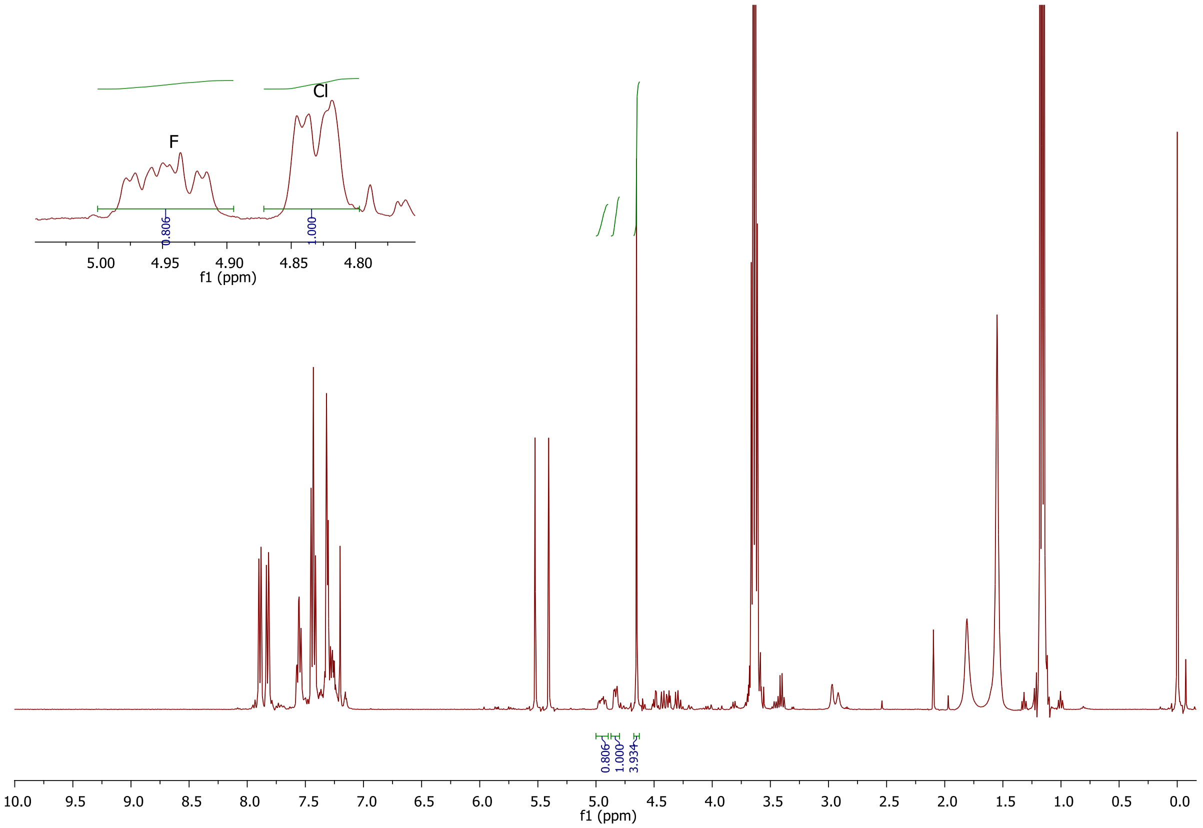
F
Cl
0.806
1.000
3.934
f1 (ppm)

60 °C

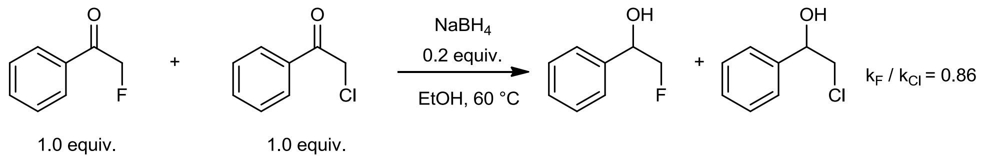

NaBH4
0.2 equiv.
EtOH, 60 °C
1.0 equiv.
1.0 equiv.
kF / kCl = 0.86

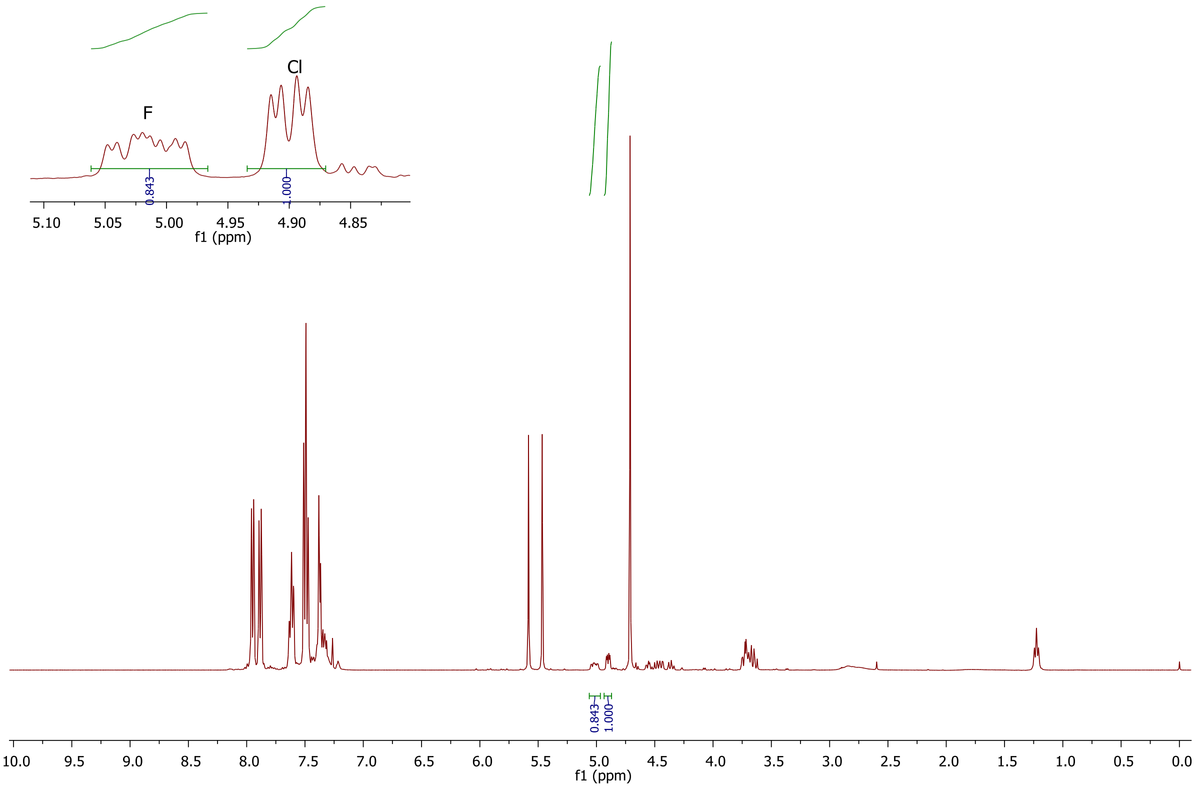

F
Cl
0.843
1.000
f1 (ppm)

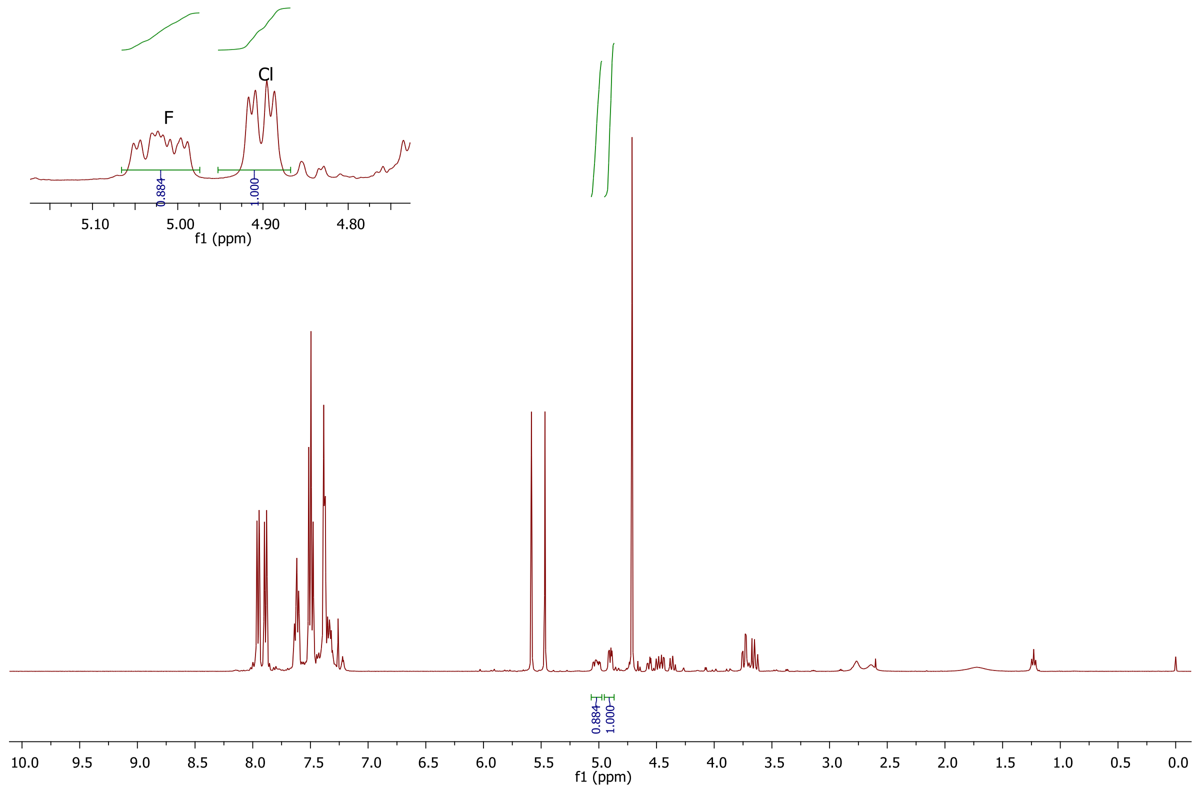

F
Cl
0.884
1.000
f1 (ppm)

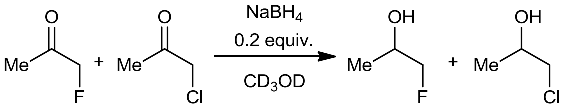

44 : 56

$k_F / k_{Cl} = 0.78$

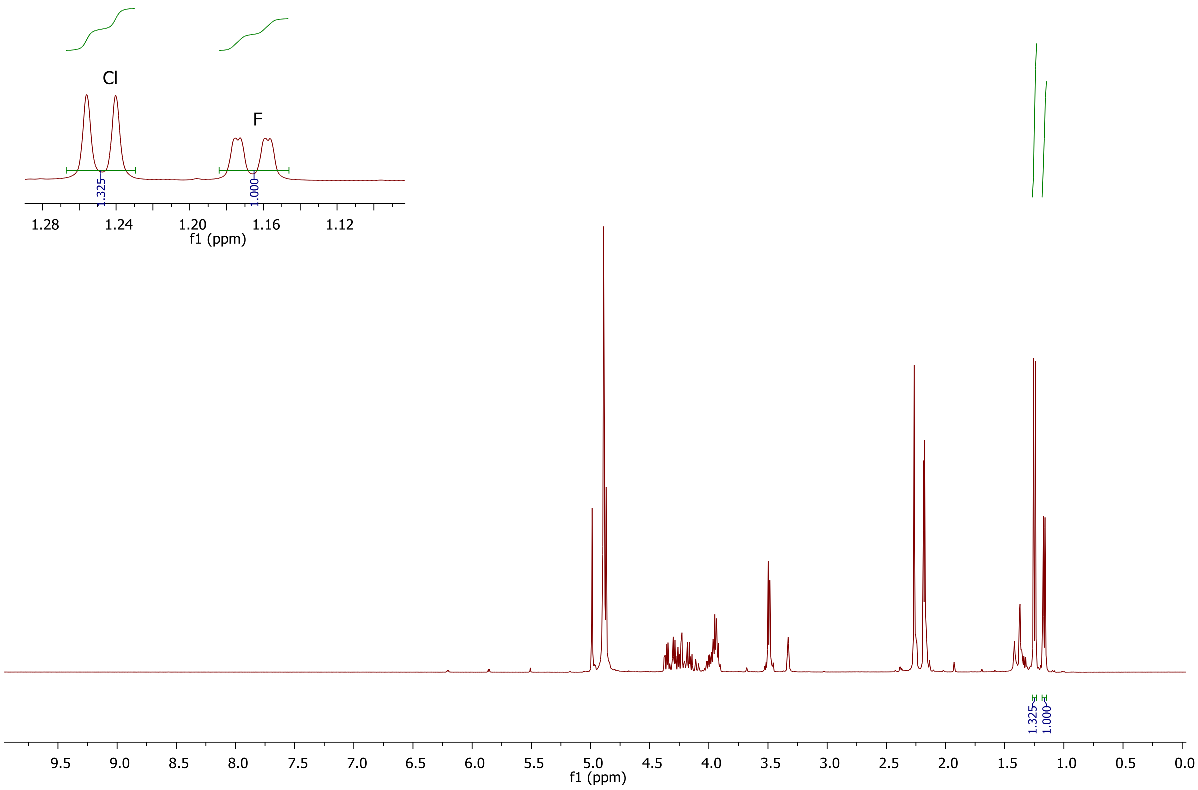

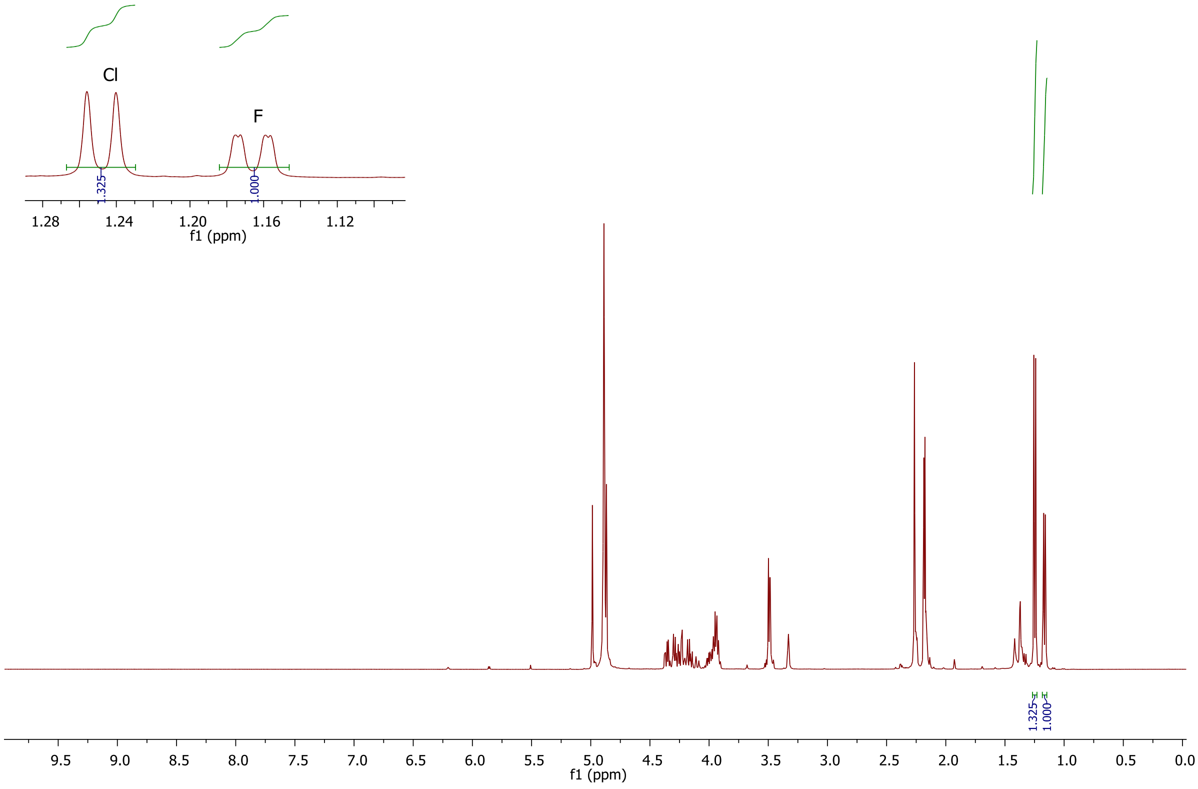

Supplement: File 1 — Copies of NMR spectra showing ratios of fluorinated and halogenated products. [file Beilstein_J_Org_Chem-13-2915-s001.pdf]
